# The role of the first level of health care in the approach to Chagas disease in a non-endemic country

**DOI:** 10.1371/journal.pntd.0007937

**Published:** 2019-12-16

**Authors:** Laura Iglesias-Rus, María Romay-Barja, Teresa Boquete, Agustín Benito, Teresa Blasco-Hernández

**Affiliations:** 1 Centro Nacional de Medicina Tropical, Instituto de Salud Carlos III, Madrid, Spain; 2 Red de Investigación Colaborativa en Enfermedades Tropicales, RICET, Madrid, Spain; Tulane University, UNITED STATES

## Abstract

**Background:**

Chagas disease has crossed South America’s borders and in recent years has spread to regions that were not previously affected. Early diagnosis and treatment of Chagas disease improves the clinical prognosis and prevents vertical transmission. Taking into account the lack of evidence of how primary care services manage Chagas disease in a non-endemic country, this study assessed Chagas disease knowledge, attitudes and practices among primary health care professionals.

**Methods and principal findings:**

Between 2017 and 2019, eight focus groups were formed with 41 family physicians and 40 nurses from healthcare centers in Madrid, Spain, and 70 field notes were collected during non-participant observation. The family physicians and nurses showed a lack of general knowledge about Chagas disease, and they did not identify the country of origin to request the blood test. The family physicians and nurses thought that the population did not talk broadly about Chagas disease because of the stigma or shame. The role of nurses was more focused on vaccination status and chronic disease follow-up, and family physicians assumed a facilitating role to send patients to different hospital facilities. Communication between primary care professionals and the hospital is a barrier frequently experienced by family physicians.

**Conclusions:**

The diagnosis of CD in non-endemic countries continues being an important challenge for health systems. The results obtained with the study of the knowledge, attitudes and practices at primary care through a qualitative approach allows to obtain evidence that could help to develop strategies for the screening of CD in a protocolized way in order to avoid that the diagnosis depends exclusively on the request of the patient.

## Author summary

Spain is the country with the highest burden of Chagas disease, with an estimated 52,000 cases, 81% of which are reported. Nevertheless, recent publications have shown that only 44% of the Bolivian population living in Madrid had a screening test performed (prevalence = 27.7%). This neglected tropical disease is transmitted through the bite of a kissing bug in endemic regions, while mother-to-child transmission, as well as blood transfusion and organ transplant transmissions occur in endemic and non-endemic countries. Without treatment, Chagas disease could cause severe cardiac and/or gastrointestinal symptoms, and ultimately death. Early diagnosis and treatment of Chagas disease improves the clinical prognosis, prevents vertical transmission and it could also prevent blood transfusion and organ transplant transmissions. Our study is the first study in Spain to use qualitative methods to explore the knowledge, attitudes and practices of primary care professionals about Chagas disease. The results show that is necessary to develop strategies in a protocolized way in order to avoid that the diagnosis depends exclusively on the request of the patient. Our findings have important public health and policy implications, offering different strategies in primary care which could facilitate the early diagnosis of Chagas disease.

## Introduction

### Context

Chagas disease (CD) is a parasitic infection caused by *Trypanosoma cruzi* (*T*. *cruzi*). CD is endemic in 21 continental Latin American countries, from southern US to northern Argentina and Chile [1]. CD is usually present in impoverished rural environments, but the internal migration from rural-to-urban areas has enabled spread to regions which were not previously affected [1,2]. Moreover, CD has crossed the continental borders because of population mobility in a way that it can be transmitted in non-endemic regions, such as North America and Europe [1]. *Triatomine insects* are the main route of transmission of *T*. *cruzi* in endemic countries, whereas congenital transmission, solid organ transplants, and blood transfusions are the predominant routes in non-endemic countries [1].

CD has an asymptomatic acute phase during which parasitaemia levels are higher [1]. This phase lasts 4–8 weeks, then enters an indeterminate chronic phase, with absence of clinical signs or symptoms of visceral involvement [1]. If untreated, the parasites could produce cardiac and/or gastrointestinal damage, causing cardiomyopathy or megaviscera in 30%-40% of infected patients [1]. Because of these complications, CD costs healthcare systems $627 million globally and leads to several disabilities [1].

The available treatments for CD are benznidazole and nifurtimox, both with different secondary effects [1]. However, recent studies showed treating infected women before pregnancy reduces vertical transmission of *T*. *cruzi*, being this the main route of transmission in non-endemic countries [1,3–5]. The efficacy is also especially high in congenitally infected newborns, with a cure rate close to 100% [4], while treatment in patients in the indeterminate phase or with visceral involvement is less efficacious [1]. Therefore, early diagnosis increases elimination of the parasite and prevents cardiac and gastrointestinal damage [1,6].

It is estimated that the prevalence of CD in the Latin American population living in Europe is about 4.2%, and the highest burden of infection is borne by Bolivian migrants [18.1%] [1,7]. Within the European continent, Spain is the country with the highest burden of CD (Chagas disease), with an estimated 52,000 cases, 81% of which are reported [8,9]. Nevertheless, recent publications show that only 44% of the Bolivian population living in Madrid had a screening test performed [prevalence = 27.7%] [10]

According to economic evaluation studies [2,11], screening for CD in the Latin American population is a cost-effective strategy in non-endemic areas, despite not being officially present [6,12]. To ensure safe blood transfusions and organ transplant, four European countries, including Spain, developed specific legislation for these procedures [6]. Nonetheless, no national strategies have been developed for screening the at-risk population, despite the flow of immigrants that Spain receives annually from endemic regions [2,6]. Some Spanish regions (Galicia, Valencia, and Catalonia) have an official protocol for primary care testing in populations from endemic areas, and some health care professionals have published guidelines encouraging screening for CD among primary care services [13]. Taking into account the lack of evidence of how primary care services manage CD in a non-endemic country, this study assessed CD knowledge, attitudes and practices among primary health care professionals in Madrid with the aim to generate a useful understanding for the design and implementation of public health initiatives and recommendations that may help strengthen interventions aimed at improving the screening and treatment of the affected population in this region.

## Methods

### Ethics statement

The Ethics Committee of Health Institute Carlos III (reference: CEI PI 05_2015-v2) approved the study. Written informed consent was obtained from all of the participants to use and publish the findings. Personal data were managed according to Spanish legislation [14].

### Setting

The present study was conducted in the city of Madrid, Spain, a non-endemic country for CD. Health care in this region consists mainly of two levels (primary and hospital care, in health centers and hospitals, respectively). The network of health centers providing primary care in Madrid is divided into seven areas of assistance from the organizational point of view, each one with its reference hospitals; however, the current law allows the population to be treated at any health center with referral to any hospital other than the reference hospital [15].

**Fig 1:**
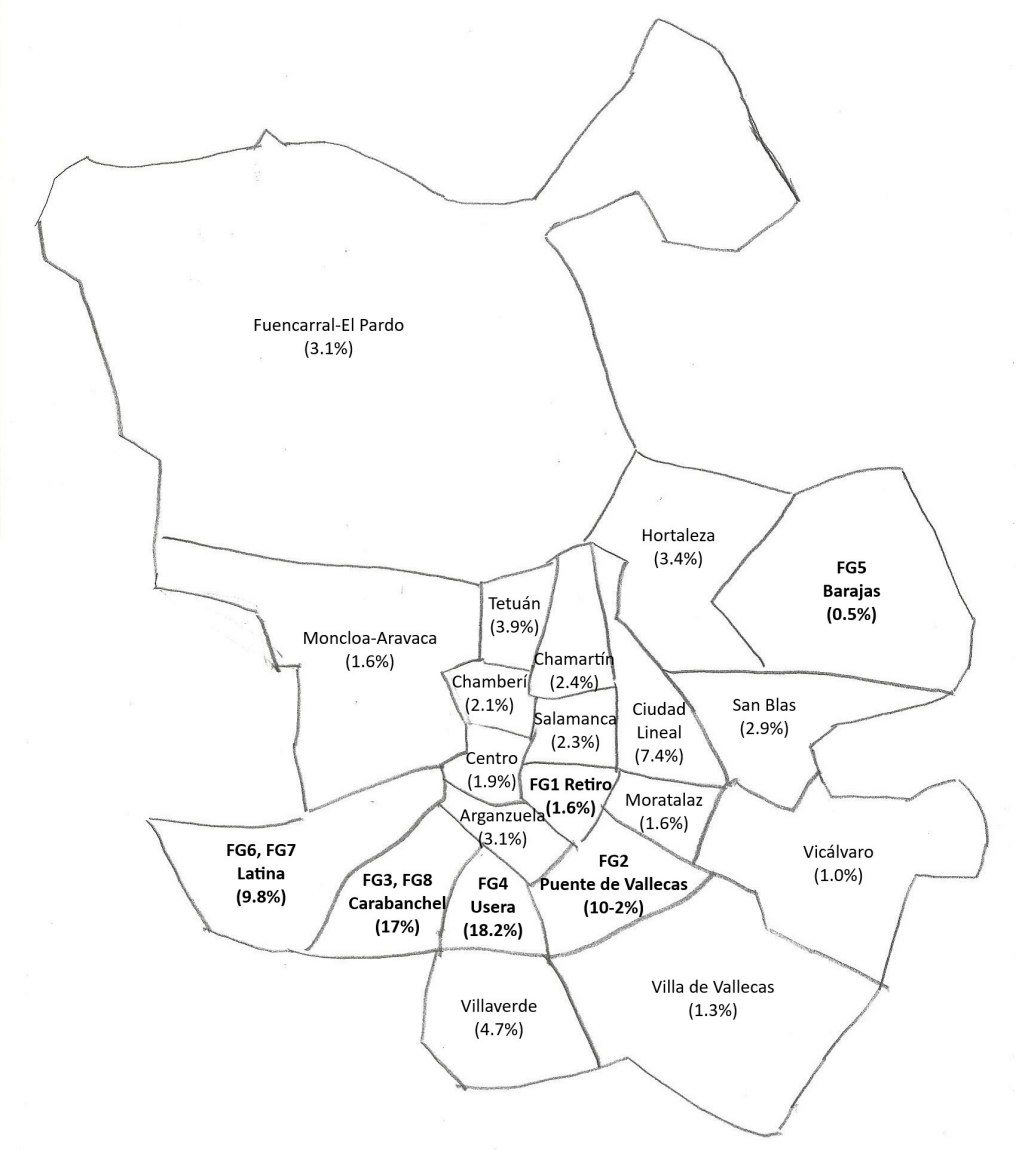
Geographic distribution of the Bolivian population in Madrid according to the municipal census and FGs. Data from https://datos.madrid.es/portal/site/egob/. [10].

The selection of the health centers where these professionals worked was based on the presence or absence of the Bolivian population in that area, according to the available data of the municipal census on 1 January 2017. Based on these data, a total of 15,591 Bolivians (6,758 men and 9,193 women) lived in Madrid, distributed principally in the municipal districts of Usera, Carabanchel, Puente de Vallecas, and Latina (Fig 1).

### Study design

The study used a qualitative research design comprised of non-participant observation and focus groups (FGs) as the main data collection techniques. To ensure that interviewers complied with the objectives of the study, a topic guide was designed; however, the guide allowed deeper exploration of all the themes that emerged during the FGs.

This study was part of a project aimed at evaluating access and use of healthcare services among the Bolivian population in Madrid. A previous questionnaire was used to collect factors related to knowledge, attitudes and practices of Bolivian population regarding management of CD, care itineraries, screening and treatment [10]. This study presents the results of FGs performed with healthcare professionals regarding their knowledge, attitudes and practices related with CD.

### Participant recruitment

The sample was qualitative and intentional. According to the types established by Teddlie and Yu [16], the sample was stratified according to the variable, “health care professionals who work in the first level of the public healthcare system,” giving rise to two professional categories: 1) family physician; and 2) nurse. New participants were recruited until no relevant new information was provided from additional interviews [i.e., the point of saturation was reached] [17].

Of the eight FGs, seven were mixed (comprised of family physicians and nurses), and one consisted of nurses alone because of the lack of point of saturation of this professional group.

Two FGs were held in municipal districts with the lowest percentage of Bolivians (Barajas 0.5% and Retiro 1.6%), and six FGs were held in districts with the highest percentage of Bolivians (Latina 9.8%; Puente de Vallecas 10.2%; Carabanchel 17%; and Usera 18.2%).

Health professionals were contacted through the medical coordinator of the primary health center, who selected the professionals based on the variables indicated by the research team.

### Data collection

The research team reviewed the FG guide to assure the potential to elicit a discussion and comprehensibility according to the Kvale model [18]. This topic guide was also modified in relation to the following FG transcripts during data collection to identify themes that required more details or new questions in subsequent FGs (https://doi.org/10.6084/m9.figshare.10295369 [PROTOCOL DOI]).

The topic guide included the following core themes: 1) knowledge of CD (transmission, symptoms, treatment, and care itineraries); 2) attitudes towards CD; and 3) practices in the consultation of the health center in relation to CD (organization, how the population goes, and what difficulties exist).

Digital recorders were used for FGs. Moreover, sociodemographic variables (sex, age, and experience working with a migrant population) were collected to give a general idea of the participants. These data were written down on paper during the FG and later entered into a spreadsheet to assure confidentiality. The FG sessions lasted an average of 60 min, had a size that ranged from 8–14 participants in each group, and took place in the health centers. Researchers worked in pairs in FGs, with one researcher serving as a moderator and the other as the note taker. Moreover, non-participant observation was conducted from July 2017 to March 2019 in the most accessible scenarios for the researchers, collecting relevant information based on the objectives of the study. Observations were made before and after all the FGs that were part of the study, on the days of the Chagas training requested by the health professionals and some sporadic observations in the halls and waiting rooms. An accurate and detailed field note record was used as an information collection instrument. All research was conducted in Spanish. Debriefing sessions were held between researchers to ensure identified themes and quality assurance.

### Data transcription, translation, and analyses

After the recording, audio files were transferred to a computer. FGs were transcribed by an external expert, and subjected to quality control by listening to the recording again to correct any errors. A thematic, inductive analysis was performed by two researchers according to grounded theory [19]. First, the transcripts were coded by line, following an inductive approach creating emergent codes summarizing the content of each sentence or paragraph. Codes and groups were further examined to look for thematic patterns in the data. To facilitate the coding process, the following software was used: MAXQDA 2018 (VERBI GmbH, Berlin, Germany); and Atlas.ti. 7.0. Scientific Software Development 2009 (Gmbh, Berlin, Germany). This thematic analysis was performed separately by the two authors involved in the analysis of the non-participant observations and FGs. Joint meetings were held to combine the results of the analysis. Also, the data collection and analysis procedures were discussed during these meetings. To provide concrete findings, data sources and analyst triangulations were carried out. The information gathered was verified and compared at different points by means of the field notes collected.

## Results

### Characteristics of the study population

Eight FGs and 70 field notes were collected from 1 July 2017 to 25 March 2019 involving 81 participants. Table 1 presents the number of FGs according to the professional category of the participants (family physicians and nurses); 75% of the professionals were women.

**Table 1:** Sociodemographic data of the participants.

	Participants					
	Family doctors	Nurses	Total	Women/men	Bolivian population (January 2017)	District
FG1	6	2	8	4/4.	1.6%	Retiro
FG2	5	4	9	5/4.	10.2%	Puente de Vallecas
FG3	6	4	10	9/1.	17%	Carabanchel
FG4	7	7	14	12/2.	18.2%	Usera
FG5	4	6	10	7/3.	0.5%	Barajas
FG6	8	4	12	10/2.	9.8%	Latina
FG7	5	4	9	7/2.	9.8%	Latina
FG8	0	9	9	7/2	17%	Carabanchel
**Total**	**41**	**40**	**81**	**61/20**		

With respect to the practice of primary care professionals related to CD, five themes emerged according to the healthcare process: knowledge about CD; professional opinions about patient’s/community knowledge and attitude; attitudes of primary care professionals in identifying factors for serologic testing; practices on testing and treatment; and patient care and follow-up circuits.

### Knowledge about CD

In general, family physicians reported having little knowledge about the epidemiology of CD, the symptoms, transmission mechanisms, and treatment, although their knowledge was broader than nurses; nine family physicians had a somewhat broader level of knowledge than their co-workers.

“And then, I mean, I know it exists (Chagas disease), I know that you have to have sensitivity when it comes to asking for it, but for example if you say ‘well, what, how do we treat it, what do we do, such …?’ Well …I have to confess that I have no idea where to start.”

(Focus group 3, family physician)

With respect to transmission of CD, the professionals did not mention vertical transmission, while both physicians and nurses mentioned vector transmission and relate the disease to the existence of the triatomine in the country of origin.

“The theme of the famous kissing bug, which is a vector disease, that is transmitted by these bugs that are like cockroaches (…) they were in houses that were mostly made of adobe, and I think the kissing bug is the little bug that makes its house there, right? or it is more usual in that area, in that type of construction.”

(Focus group 1, family physician)

Family physicians had knowledge about the relationship between CD, poverty, rurality in the country of origin, immigration, shame of the patient related to the disease, and the low efficacy of treatments.

“Because as far as I know, there are no great effective treatments, then diagnose a person of something that does not have effective treatment, which has a very long course, with an evolution very deadly or very asymptomatic …”

(Focus group 1, family physician)

The nurses, on the other hand, associated CD with South America. However, they expressed vague knowledge about CD, and frequently mistakenly associated CD with the Zika virus.

“I think I remember how 3–4 years ago there was an outbreak of this disease, (…) they put posters on the door and especially emphasizing pregnant women (…) maybe I'm mistaken of disease, but I do not think so. It had something to do with the microcephaly that occurred in newborns.”

(Focus group 8, nurse)

The family physicians who showed better knowledge about CD justified the following reasons: 1) participation in research studies or contact with specialists in Infectious diseases and Tropical medicine; 2) those who worked in areas with a greater population coming from an endemic area of CD indicated improved knowledge from contact with positive cases or with patients who expressed the need to be diagnosed; and 3) some of the health professionals justified differences in knowledge by mere personal concern and by being born in Latin American countries.

“Well, when I arrived at the health center, a study was being conducted in the hospital about Chagas disease. Then some doctors participated (…) and consisted of patients who were part of the countries where there was a tendency to Chagas, you had to pass an informed consent and you had to ask for a Chagas serology …”

(Focus group 7, family physician)

"And then, apart from that, patients have been coming to me, some because they had already been diagnosed of Chagas disease in their country, and then they came to tell how long they had not controlled themselves, and others commented ‘Well, look, I was fine but, as in my town there are people with Chagas, I feel that I have the same symptoms’."

(Focus group 7, family physician)

“Well, I’ve been here for 17 years and I studied it (Chagas disease) in my country. In third grade in Parasitology, they taught us about the parasite that produces it (…) esophageal achalasia, megaesophagus, chagasic cardiomyopathy (…) I am from Ecuador.”

(Focus group 4, family physician)

Family physicians commented that in spite of having received some specific training in tropical diseases, their continuous training is focused on chronic pathologies that are frequently encountered in consultation, such as diabetes or high blood pressure.

“The problem is what happens to us with other diseases or with management of other health problems: if they are not frequent or habitual, in the end what you learn one day (…) is forgotten. It is impossible to be updated and handle all pathologies. In the end you update and handle the most usual and most frequent. But other things …it's complicated.”

(Focus group 5, family physician)

Nurses expressed that learning about tropical diseases comes from a specific case that presented in consultation.

“As the nurse has to know everything, once tropical disease comes to you and you face it, you form yourself about it, but it does not fit into our daily training.”

(Focus group 8, nurse)

Both physicians and nurses, in the absence of knowledge about CD, manifested a proactive attitude, requesting training sessions after conducting the FGs, and began to actively seek out the population from endemic areas.

“5 June 2018. Health center. One of the professionals proposes to his colleagues to search people from South America after the FG.”

(Field note, observation)

“15 December 2017. Health center. A family doctor proposes to the researchers to give a clinical session about Chagas disease for all the Primary Care team.”

(Field note, observation)

### Professional opinions about patient’s/community knowledge and attitude

Family physicians believe that the patient has a dual perception of CD: those who express concern about CD, the symptoms, death, sick or deceased relatives, and the possible involvement of children; and those who assume a predetermined evolution and manifesting little awareness of CD.

“Maybe there (in Bolivia) they are surrounded by people with Chagas disease, by people that dies of heart disease with 40 years old (…) So maybe the attitude they have is to normalize the disease, right? Maybe they think Chagas disease is something usual because their brothers or their grandfathers died, their mothers have it…” “I think they are people who feels okay (asymptomatic), so they do not have a sickness concept.”

(Focus group 2, family physician)

In addition, professionals pointed out that patients coming from an endemic area do not usually talk about CD with their family physician; however, family physicians and nurses have the feeling that patients talk about it among their peers within the community.

"You even ask … people from Bolivia if there are cases in the family and they change facial gesture … I do not know if … they do not know what it is or if they do not want to tell you."

(Focus group 6, family physician)

In relation to the stigma associated with CD, some nurses indicate that the disease is treated as taboo by the affected people. In the case of family physicians, they have two different perspectives: those who consider that there is no stigma related to the disease; and those who believe the stigma is not linked to the disease, but to poverty and being immigrants.

"And I was not aware that (…) it's a stigma. (…) I was not aware that it is associated with poverty. So, of course, if I do not even remember, I'm not aware and I do not ask him anything (about it)…"

(Focus group 7, family physician)

“Well, I think it's different there than here, maybe there it's more related to poverty, to adobe houses, to very rural things and a lot of poverty, and here more to immigration. Well, it's a disease of immigrants, and maybe it's more in that way than because of extreme poverty, right? A disease of people brought from the outside."

(Focus group 2, family physician)

Finally, there was disagreement between professionals about the existence of access barriers in diagnosis and follow-up for the population from an endemic area of CD: some professionals explain that the main impediments that can be found are related to the social problems of this population group instead of the difficulties of the health system organization, whereas few professionals pointed out that their lack of knowledge is already an access barrier to health care.

"I have patients who rarely come here because of the work they have …And then, (…) when they come (to health center), maybe they come once a month or every two months, with problems that we try to solve at the time. Things (other diagnostic tests) are requested, they do not get them, and they come back with the same problems unsolved again. "

(Focus group 5, family physician)

"So the first barrier that I have found is that we do not know the nationality of our patients (…) but regardless of whether you know the disease, you do not … that is, you do not place your patient in an environment, then it is difficult to diagnose something if first you do not know very well … "

(Focus group 2, family physician)

### Attitudes of primary care professionals in identifying factors for serologic testing

In reference to the country of origin, not all family physicians know if the population they work with comes from an endemic area. In addition, family physicians have a tendency to encompass the entire Latin American population as a homogeneous group, identifying them fundamentally by phenotype.

"They look like their parents. They have (physical) features and you already know that maybe they are not Spanish. That maybe they are from … and then you ask them for sure, you know? Unless if she is a blonde, blue-eyed girl … you do not even think about it. And maybe it turns out that the mother is, I do not know, she is whiter, she is not so indian, and she has lived with another population. But, well, the physical aspect, at first…"

(Focus group 7, family physician)

"But it is also clear that I do not know those cultures very well, I mean, for me they are all … they come from South America and that is all, and they are … yeah, it’s wrong"

(Focus group 3, family physician)

In the case of very established population or people who are born in Spain, family physicians do not think about the possibility of a family history of CD, the origin of the patient’s parents, or the existence of suggestive symptoms.

“People who comes to (medical) consultation is people who has been coming for a long time, I mean (…) they are settled, very established here…”

(Focus group 2, family physician)

In addition, family physicians have referred to the concept of "healthy immigrant" on several occasions; physicians were concerned about their pathologies here instead of the prevalence of migrants’ origin country.

“But the message that was left at that time was that immigrant patient is a man whose pathology is not brought here and give it to everyone else; he gets sick of the local diseases, that was the idea…”

(Focus group 1, family physician)

Both physicians and nurses claim that the interest in the country of origin is more focused on the concern for social problems and vaccination status, but it is not consistently recorded in the medical history.

"I do often ask it, as a nurse, but the truth is I am not thinking about any disease but rather thinking about … well, the anxiety that comes from being here, about the social thing, if he has the family there, if he has it here, how is it going, how long it takes … "

(Focus group 2, nurse)

Specifically, professional groups recognize that part of their Spanish elderly population is cared by the population born in Latin America, although they do not identify the susceptible needs of the latter beyond their role of caregivers.

"And there are many (women) who are taking care of patients and, in fact, many times I do not even know they are patients of mine. And someday she appears in the consultation, and, oh, ‘you are my patient’! 'Yes, Yes. But I could not come because I was with the elderly and such.' So … "

(Focus group 5, family physician)

### Practices on testing and treatment

In reference to the behaviors of the professionals, both physicians and nurses acknowledge their low suspicion of CD and pointed out that is not the norm to request a serologic determination from primary care, either due to ignorance of the possibility or due to ignorance of access from the request of the computer program.

“I’ve found out it (Chagas serology) can be requested this morning.” “In fact, I was thinking about asking how serologies are done …”

(Focus group 1, family physicians)

In a minority, some nurses referred to the possibility of requesting blood testing autonomously or under the supervision of the physician depending on the function of each primary care team.

”The family physician requests them (blood tests), although blood tests of (people with) chronic diseases can be requested by the nurses.”

(Focus group 8, nurse)

In the case of pregnant women from endemic countries, whether or not to request a blood test is related to the existence of protocols or consensus of control of vertical transmission in the reference hospitals, although these procedures are not always known by physicians and nurses in primary care. In some cases, physicians assume that the request is made by the Obstetrics service (gynecologist or midwife), although very rarely and only some physicians who work in areas where there is a greater population from Latin America requested the test.

“It depends on the area, there are some protocols that the blood test is requested by the family physician; and here for example, it is directly requested by the obstetrician when he sees her.”

(Focus group 2, family physician)

Family physicians expressed that family history is the basis for blood testing in childbearing women, like a patient request or reporting a previous positive diagnosis into her family, even though that it is not a common situation.

“She came two weeks ago, and she told me that her sister had been diagnosed with Chagas disease. She is from Bolivia, so I requested it (the blood test) (…) She did not come saying ‘I want the blood test’, but ‘Something came up, I have this doubt, maybe…’”

(Focus group 3, family physician)

On the other hand, nurses express they have not met in any patients who request diagnostic serology for CD.

“Maybe some patient has been attended or followed up here (in the health center), but not by the nurses…”

(Focus group 8, nurse)

Regarding the treatment of CD, physicians assume that treatment is not a competence of primary care, and they think that its efficacy is very low.

“About the treatment, we do not take part in anything, nor in the prescription (of the medicine)… In fact, I believe that they get it in the hospital pharmacy…”

(Focus group 4, family physician)

“They have a treatment, it is true that it has several secondary effects, and it does not cure the disease.”

(Focus group 2, family physician)

Physicians do ask about the country of origin when this information is part of a pathologic study, when recommended by other specialists, or upon close contact with specialists in Tropical diseases. Their justification for this practice is based on the possibility of annoying or offending the patient.

"I do not ask for the origin by system. Yes, if maybe, well, there is some disease, some clinical reason, uh … then yes. If not, I do not usually ask or reflect it in the (clinical) history." "Also, when you register you have to be careful, because I have had patients who have (said) … ‘Well, but why do you have to write that?’ (…) I think there should be a sensitivity when it comes to write it so that the patient does not feel … as if he was … labeled in such a way … "

(Focus group 3, family physician)

This situation changes slightly in the health centers in which there are more patients coming from an endemic area, because the professionals are most knowledgeable about CD and take into account the family history, the possible symptomatology, and the country of origin.

"Sometimes there are young patients who suggest chest pain, symptoms of cardiac dysfunction, so I ask them where they are from, and if they are from Bolivia I request the Chagas test as well."

(Focus group 4, family physician)

Finally, the nurses reported that they have limited contact with young adults and that their role in primary care with the immigrant population is mainly focused on the vaccination status of the pediatric population, the promotion of health, and follow-up of chronic diseases.

“It is that the nurses here work with pediatrics and with adults, we do not make a distinction (…) and then we work at a preventive level in the reviews of the child (…) and then the chronic patient is managed by the nurse.”

(Focus group 8, nurse)

### Patient care and follow-up circuits

According to family physicians, the practices in relation to the care circuits of patients with CD are very heterogeneous because of the characteristics of the referral hospital or because of the services with which the professional is familiar. It is important to note that the physicians stated that the patient with CD is not always sent to the referral hospital or to the same medical specialties, although they show a predilection for the Tropical Medicine units, even when they are far away from the health centre.

“Well, it depends, because at the beginning we usually derived to Infectious Diseases services of another hospital (…), and then (during) one season to Tropical Diseases from another hospital. But now we have an Infectious Disease service in our hospital, and … I mean, we can refer them directly (to those specialists) or through Internal Medicine"

(Focus group 4, family physician)

In addition, those family physicians who are unaware of the possibility of requesting diagnostic testing from primary care refer the patient directly to the hospital services without performing blood testing.

“I have had two or three cases, but I referred them to hospital, I did not know it, I am learning that we can ask for the (blood) test ourselves."

(Focus group 1, family physician)

On the other hand, those patients who come with a previous diagnosis are referred to the hospital specialists, so family physicians claim to assume a facilitating role.

"He came from … yes, it's true, he came from South America, he had just arrived to Spain, and then he had just been newly diagnosed with Chagas disease. Then he went to the consultation … he had … his brother, he had a brother, a sister, I cannot remember, and he came to my consultation in order to send him to assess follow-up."

(Focus group 3, family physician)

In the case of pregnant women, in those health centers sited in areas of Madrid where there are more patients coming from an endemic area, they affirmed the existence of specific referral and coordination circuits established by the referral hospital. In these cases, the midwife or the family physician requests a serologic determination prior referral to a gynecologist.

"You have the option if you want to request the protocol of the first trimester, but usually what we are doing and what they (Obstetrics service) recommend is to go to the midwife, the midwife receives her and requests everything so that things (relevant information) are not lost … so I think that the major work is done by midwives (…) And they make the assessment of whether they have criteria to request (blood test for) Chagas disease or not."

(Focus group 3, family physician)

However, in the case of women of childbearing age, no mention was made of the possibility of screening this population.

On the other hand, nurses showed doubts or ignorance about their role in the process of attention to CD.

"Anyway, there is a question that comes to me about all this and it is: you have this kind of patient. . . .and what would you do as a nurse in those cases? (…) Would be a patient who in some occasion or in some process of his pathology should be referred to nursing?"

(Focus group 6, nurse)

In addition, the nurses assume that the pregnancy is only monitored by midwives.

“We have a midwife, so when the pregnancy is detected, it is referred to the midwife, and she is the one who usually makes the first blood test”

(Focus group 8, nurse)

With respect to the follow-up, family physicians comment that patients do not usually return to the primary care consultation for CD follow-up when diagnosed, although the reasons for coming back usually focus on other reasons for consultation or on a request for new referrals due to the inability to attend hospital appointments.

"I have had 3 patients. One was a pregnant woman and the others were something that started as a result of a cardiac clinic, but it was not here, it was in other area. And … one of them, what I do remember is that she was diagnosed and disappeared. She returned after 2 or 3 years without doing anything, she did not go to the (hospital) consultation.”

(Focus group 6, family physician)

In addition, family physicians indicate that the flow of information between the hospital and primary care is erratic, depending mainly on the presentation of reports by the patient due to the difficulty to access hospital information.

"We do not have access to the hospital (information), so he has to bring the written report that he has gone to the cardiologist and they have seen the heart disease, but if they do not bring that report, the patient tells me everything is fine. And that's what you have, and that's what you write."

(Focus group 1, family physician)

In particular, family physicians admit having a poor participatory role in the follow-up of CD, assuming complete monitoring by hospital services and demanding little information about the process.

"I saw them in another health centre, but I saw them from the family (physician’s) consultation, (…) it was not because of the Chagas problem. They had their follow-up of Chagas in the Infectious service of the hospital and they came to the normal family (medicine) consultation and well, I could ask them, but it was not a matter for me specifically following Chagas. Well, more symptomatic (treatment) and so if there were symptoms, if not …"

(Focus group 6, family physician)

## Discussion

The results show the lack of knowledge about CD, the epidemiology in a non-endemic country, and the heterogeneity of practices of primary care professionals in the management of this health problem.

In general, professionals consider the main route by which CD is transmitted involves vector, creating a representation of the disease centered on the bite of the triatomine. In addition, the possibility of vertical mother-to-child transmission is not clearly identified because the idea that the father can also transmit the disease is contemplated. There is also a lack of familiarity with the possibility of requesting the serologic test from the primary care consultation. Other studies conducted in non-endemic countries [20–23] also suggest the lack of knowledge of health professionals about CD in non-endemic areas. The reason why health professionals lack training related to CD despite having the available diagnostic tools is not known, although it could be that the training in tropical diseases provided by national health system is perceived as a training that has little applicability in clinical practice.

The country of origin was not taken into account by health professionals as a possible risk factor for certain health problems, assuming that the health status of the migrant population is optimal [24]. Nevertheless, some authors point out the susceptibility to a deterioration in health status of the migrant population that is influenced by the migration process, working conditions, socioeconomic status, and access to health services [24].

This situation relegates the diagnosis of CD to a patient request. Indeed, the patient decides whether or not to communicate personal and family histories to the health professional; blood testing also depends on the skills and knowledge of the health professional performing the evaluation. According to recent studies conducted in Madrid with Bolivians, performing diagnostic testing is at the discretion of a physician and the advice of a family member or at community campaigns [10].

In addition, the diversity of practices among different professionals in the diagnosis and monitoring of CD is reproduced at the national level, taking into account the different existing screening programs in the different regions of the Spanish National Health System in the absence of a national consensus protocol [12,25]; however, these programs are focused on the prevention of vertical transmission, so that the rest of the population at risk is not included [13], despite the broad trajectory of Latin American migration in Spain and the economic studies that indicate that both the screening of CD in asymptomatic adults and the control of pregnant women, newborns, and first and second degree relatives of Latin American origin are cost-effective strategies [2,11].

Specifically, primary care in Madrid does not seem to participate nor have a leading role in the process of diagnosis and follow-up of CD, thus relegating these functions to hospital services. Taking into account that health centers are the health resource closest to the population and the response capacity of family and community medicine due to their adaptability and generalist nature [26], primary care services are in an advantageous position to manage CD. In addition, the scarce role of Spanish primary care nurses with respect to CD needs special attention because according to the competences regulated at the national level, "family and community nursing acquires a special commitment to the most disadvantaged social sectors for reasons of social class, gender, ethnicity, age, disability, illness, etc. " [27].

Moreover, the coordination of health centers with tropical disease referral centers would allow the early detection of asymptomatic CD and visceral involvement, and the offer of treatment and follow-up of the pathology for the prevention of complications and risk factors related to lifestyle [1,6,28]. The published consensus about the approach to CD in primary care [13] facilitates the detection and management of CD by primary care physicians, basing their recommendations on the correct evaluation of epidemiologic risk, the anamnesis of signs and symptoms of organic involvement, and the performance of complementary tests for diagnosis and monitoring [13].

### Strengths and limitations

One of the strengths of the study was that this is the first study conducted in the field of primary care in Madrid regarding the management of CD by health professionals.

Being a study conducted in the city of Madrid, the present findings may not be generalizable in other contexts, although the findings do provide information on possible areas of improvement in the care of CD in non-endemic areas.

### Conclusions

The diagnosis of CD in non-endemic countries continues being an important challenge for health systems. The results obtained with the study of the knowledge, attitudes and practices of the health care professionals at primary care through a qualitative approach allows to obtain evidence that could help to develop strategies for the screening of CD in a protocolized way in order to avoid that the diagnosis depends exclusively on the request of the patient.

### Recommendations

Our study findings support the following recommendations to the health care system:

- Training and mentorship of primary care professionals on CD and its treatment;
- Create consensus, unified documents, and protocols for CD control in Spain;
- Improve flow of patient information between primary care and hospitals;
- Implementation of epidemiologic surveillance of CD at the regional and national levels;
- Strengthen the role of the family and community nurse for the opportunistic recruitment of the population;
  ○ To include the serologic request to those girls born in an endemic area or whose mothers came from an endemic area in the scheduled reviews of girls 12–14 years of age according to childhood care protocol;
  ○ Offer CD screening to women of childbearing age from endemic area when they request cytology or mammography;
  ○ Register the country of origin and family history routinely in patients at risk;
  ○ In the case of those patients coming from an endemic area with chronic diseases, include the diagnostic testing of CD in the control analytics
